# How Do Different Types of Entrepreneurial Networks and Decision-Making Influence the Identification of Entrepreneurial Opportunities?

**DOI:** 10.3389/fpsyg.2021.683285

**Published:** 2021-07-16

**Authors:** Weizhen Yu, Myeongcheol Choi, Jianzhuang Zheng

**Affiliations:** ^1^School of Management, Zhejiang Shuren University, Hangzhou, China; ^2^Department of Global Business, Gachon University, Seongnam-si, South Korea; ^3^School of Business, Zhejiang University City College, Hangzhou, China

**Keywords:** entrepreneurship, entrepreneurial network, decision-making, entrepreneurial opportunity, entrepreneurial firm

## Abstract

Entrepreneurial networks are important for the identification of entrepreneurial opportunities and development in the context of social media. This exploratory research investigates the relationships in entrepreneurial networks, by considering decision-making, and entrepreneurial opportunities, and focusing on the role of decision-making in the relationship between entrepreneurial networks and entrepreneurial opportunities. Using data from 512 Chinese entrepreneurial firms, hierarchical regression analyses and structural equation modeling are employed to create a mediation model that links entrepreneurial networks to entrepreneurial opportunities through decision-making. Our findings are as follows: (1) heterogeneous networks are positively related to innovative opportunities, and homogeneous networks are positively related to imitative opportunities; (2) heterogeneous networks positively affect non-linear decision-making (non-linear DM) while homogeneous networks positively influence linear decision-making (linear DM); (3) positive relationships exist between non-linear DM and innovative opportunities and between linear DM and imitative opportunities; and (4) non-linear DM fully mediates between heterogeneous networks and innovative opportunities, and linear DM partially mediates between homogeneous networks and imitative opportunities. This paper contributes to literature on entrepreneurship by broadening understanding of the mechanisms of entrepreneurial opportunity formation in emerging markets and provides important insights for entrepreneurs and policymakers.

## Introduction

With the use of social media, entrepreneurs can further invigorate China's economy. There is evidence that the growth of new ventures not only increases economic activity, but also promotes a country's economic structure and the quality of its development, which then becomes the driving force for economic transformation (Petuskiene and Glinskiene, 2011). In particular, entrepreneurs are taking advantage of different ways of spreading business ideas and gaining useful information and advice from their networks. Stimulated and supported by government policies and the market, more and more people are becoming entrepreneurs. However, in strong contrast to high activity, a high failure rate is sometimes observed.

Opportunity is an essential element of entrepreneurship. It is “a situation in which a person can exploit a new business idea that has the potential to generate profit” (Shane, 2003). Generally, an entrepreneurial opportunity may lead to the formation of new economic activities and new organizations and is key to a start-up's success or failure (Foss et al., 2013; McMullen and Dimov, 2013; Davidsson, 2015). Compared with developed countries, most of the entrepreneurial opportunities in China are based on the diffusion and application of existing knowledge, focused on imitative opportunities for technology and market defects (Shane and Venkataraman, 2000; Di Muro and Turner, 2018). Therefore, exploring the formation mechanism of different entrepreneurial opportunities in a dynamic environment and identifying possible paths of high-quality entrepreneurial opportunities that correspond to the attributes of entrepreneurs are not only important academic issues for building and understanding entrepreneurship, they are also necessary for assisting and guiding entrepreneurship activities (Chen, 2019; Feng and Chen, 2020).

One of the most crucial debates in entrepreneurship research is whether new business opportunities are created or discovered by entrepreneurs. Studies on the identification of entrepreneurial opportunities mainly focus on two different epistemological assumptions, namely, the Schumpeterian and Kirznerian views (Suddaby et al., 2015). Schumpeter (1934) stressed the role of the entrepreneur as an innovator who “shocks” the economic equilibrium during times of uncertainty, change, and technological upheaval and found that entrepreneurial opportunities are created in the economic system. However, Kirzner (1973, 1997), with an opposite view, stated that the discovery of opportunities is the core issue of entrepreneurship. In reality, entrepreneurial networks are the key variable in opportunity creation or discovery, especially in emerging markets like China. Moreover, abundant evidence shows that the generation of new opportunities and ventures is attributable to business networks (Aldrich and Cliff, 2003; Tamasy, 2006). China's economic transition has increased the uncertainty of entrepreneurial activities, and entrepreneurial networks could be crucial for managing the success of entrepreneurship.

This study examines whether the contributions of entrepreneurial decision-making and networks to entrepreneurial opportunities are key factors in the mechanism of entrepreneurship. All behavior can be seen as products of the simultaneous operation of two different types of information processing methods: linear and non-linear thinking (Kahneman, 2003). Specifically, linear thinking includes objective, continuous, convergent, constrained, logical, critical, and detailed rational thinking, while non-linear thinking includes subjective, divergent, unconstrained, integrated feelings and overall creative thinking. Moreover, every action serves as a network's main body and “node.” Although many scholars suggest that entrepreneurial networks are key antecedents of the identification of entrepreneurial opportunities, the relationships among entrepreneurial networks, decision-making, and opportunities have not been explored in full to date. This study explores the matching of different types of entrepreneurial networks and decision-making to entrepreneurial opportunities.

This exploratory research develops a mediation model to highlight the importance of decision-making in the linkage between entrepreneurial network and entrepreneurial opportunity, and answers the following questions:

1. Do entrepreneurial networks influence entrepreneurial opportunity?
2. Do entrepreneurial networks influence decision-making?
3. Does decision-making influence entrepreneurial opportunity?
4. Does decision-making play a mediating role in the relationship between an entrepreneurial network and opportunity?

This study responds to the need for entrepreneurship research in the context of social enterprises by providing new evidence of the relationships among entrepreneurial networks, decision-making, and entrepreneurial opportunities. Based on a large sample of Chinese entrepreneurial firms, we broaden the understanding of the mechanisms of opportunity formation in emerging markets. Our findings can help entrepreneurship researchers understand how different types of entrepreneurial networks and decision-making styles affect the identification of entrepreneurial opportunities. Lastly, this paper contributes to existing entrepreneurship literature and provides important insights for entrepreneurs and policymakers.

The rest of this paper is structured as follows. Literaure review and hypotheses and development presents an overview of the literature and discusses the research framework, along with the proposed hypotheses. Methodology presents the research methodology and describes the sample, data, and variables. Analysis and results provides estimations of the proposed effects and illustrates the results. Discussion and implications discusses the theoretical and practical implications of this study and addresses its limitations and directions for future research.

## Literature Review and Hypotheses Development

### Entrepreneurial Networks and Entrepreneurial Opportunities

Entrepreneurial network*s* refer to the channel*s* and carriers of information, knowledge, and resource exchange, and interaction. These networks are important for entrepreneurs to deal with the uncertainty, urgency, and pressure of identifying entrepreneurial opportunities, and reduce transaction costs (Ardichvili et al., 2003; McMullen and Shepherd, 2006). Therefore, entrepreneurial networks are not only “sources” of information, but also serve as “bridges” that help improve the timeliness, relevance, and quality of the information obtained, and are a determinant of entrepreneurial opportunities (Adner and Kapoor, 2010; Cantù, 2018).

Many scholars have argued that high-quality entrepreneurial networks help entrepreneurs identify potential opportunities (Davidsson and Honig, 2003; Hoang and Antoncic, 2003). In reality, most entrepreneurs tend to use business networks to obtain both internal and external information and resources, thereby broadening their thinking modes and decision-making, and promoting the flow and allocation of related resources that can help identify entrepreneurial opportunities (Rosenbusch et al., 2011; Cardon et al., 2017). For every start-up, identifying the network characteristics that match the start-up beforehand is important for building an active network, because choosing the right entrepreneurial network is a make-or-break decision for successful entrepreneurship.

To further explore the effectiveness of properly matching entrepreneurial networks and opportunities, this study classifies entrepreneurial opportunity into two types—innovative opportunity and imitative opportunity. An innovative opportunity (also referred to as Schumpeterian opportunity) is an opportunity that breaks away from existing routines, whereas an imitative opportunity (also referred to as Kirznerian opportunity) is an opportunity to build incrementally upon, or replicate, an existing business, product, or service (Shane, 2003; Holmén et al., 2007; Samuelsson and Davidsson, 2009; de Jong and Marsili, 2015). Based on existing literature (e.g., Beckman and Haunschild, 2002; Nieto and Santamaría, 2007), we created two measures of entrepreneurial networks: heterogeneous and homogeneous. We conjecture that a network that has heterogeneous actors and generates diverse sources of information and knowledge enables entrepreneurs to identify a novel opportunity effectively. Meanwhile, collaborations within a homogeneous network facilitate interaction with other actors to gain greater professional experience, which impacts the opportunity for entrepreneurs to identify development opportunities. Based on this analysis, the following hypotheses are proposed:

*H1. Entrepreneurial networks are positively related to entrepreneurial opportunities*.
*H1a. Heterogeneous networks are positively related to innovative opportunities*.
*H1b. Heterogeneous networks are positively related to imitative opportunities*.
*H1c. Homogeneous networks are positively related to innovative opportunities*.
*H1d. Homogeneous networks are positively related to imitative opportunities*.

### Entrepreneurial Networks and Decision-Making

Different thinking styles lead to different decision-making strategies, and the mode of thinking is a learned cognitive process for sensing, understanding, and predicting external events, which also interacts with the external environment to optimize the actors themselves (Groves et al., 2008; Vance et al., 2008). Entrepreneurial cognition may vary depending on the unique circumstances and requirements of a particular situation (Dane and Pratt, 2007). At the same time, both social information processing theory and interpersonal attraction theory state that the formation of entrepreneurial networks affects the cognition of entrepreneurs and thereby influences their decision-making (Kamm and Nurick, 1993; Jansen et al., 2013).

Two studies have influenced the development of this study's hypothesis. Beckman and Haunschild (2002) stated that “one important attribute of network structure that can affect decision quality is partners' heterogeneity.” Entrepreneurs broaden their vision and reach decisions similarly when they are part of a heterogeneous network. Meanwhile, Ruef et al. (2003) proposed that entrepreneurial founding teams are likely to emerge in a homogeneous network. A possible reason is that approaching homogeneous network members that have similar characteristics greatly influences entrepreneurs' decision-making or motivates entrepreneurs to make decisions. As previously mentioned, information processing can be linear or non-linear (Kahneman, 2003), and this applies to decision-making, which can be non-linear decision-making (non-linear DM) or linear decision-making (linear DM). Therefore, the following hypotheses are proposed:

*H2. Entrepreneurial networks are positively related to decision-making*.
*H2a. Heterogeneous networks are positively related to non-linear DM*.
*H2b. Heterogeneous networks are positively related to linear DM*.
*H2c. Homogeneous networks are positively related to non-linear DM*.
*H2d. Homogeneous networks are positively related to linear DM*.

### Decision-Making and Entrepreneurial Opportunities

The way that entrepreneurs make decisions depends on whether they believe that what they are seeing is an opportunity (Shane and Venkataraman, 2000). In the literature, the key arguments that support the concept of opportunity creation or discovery appear to involve different decision-making modes (Maine, 2015). Furthermore, decision-making is usually generated by the combined influence of linear and non-linear thinking styles, thereby leading to an intrinsic psychological plan that guides problem-solving (Vance et al., 2007; Zhang et al., 2010).

In a dynamic environment, markets change incredibly fast. This, plus large amounts of data, environmental information, and other inputs, require immediate non-linear DM. However, a linear approach in terms of logic, analysis, and verifiable data can also aid in solving business challenges. Nonetheless, entrepreneurs with non-linear thinking and decision-making styles are more likely to grasp promising business opportunities (Shane and Nicolaou, 2015). In this study, we conjecture that entrepreneurial opportunities are identified via both linear and non-linear decision-making. Therefore, the following hypotheses are proposed:

*H3. Decision-making is positively related to entrepreneurial opportunities*.
*H3a. Non-linear DM is positively related to innovative opportunities*.
*H3b. Non-linear DM is positively related to imitative opportunities*.
*H3c. Linear DM is positively related to innovative opportunities*.
*H3d. Linear DM is positively related to imitative opportunities*.

### The Mediating Role of Decision-Making

With the development of social media, entrepreneurial networks are able to provide entrepreneurs with a wealth of information and increase the probability of opportunity recognition (Drummond et al., 2018). However, the reception of information differs from the acceptance of information. Entrepreneurs use their own cognitive styles to connect information and generate new understandings of the internal structure of things by reconnecting or combining information in different ways (Ozgen and Baron, 2007). The creation of new ideas stimulates information exchange that helps in identifying entrepreneurial opportunities and leads entrepreneurs to believe that what they are “seeing” is an opportunity (Campos et al., 2015). Diverse decision-making styles (resulting from different ways of thinking) may lead to different responses by entrepreneurs who have the same information; hence, all these factors combine to affect the identification of forward-looking information as entrepreneurial opportunities.

During the process of opportunity identification, entrepreneurs have to use their unique ways of decision-making to improve the quality of entrepreneurial activities. From a causal logic perspective, decision-making is influenced by entrepreneurial networks; at the same time, it also affects the opportunity formation process. Therefore, we conjecture that decision-making mediates the relationship between entrepreneurial network and entrepreneurial opportunity, and propose the following hypotheses:

*H4. The relationship between entrepreneurial networks and entrepreneurial opportunities is positively mediated by decision-making*.
*H4a. The relationship between heterogeneous networks and innovative opportunities is positively mediated by non-linear DM*.
*H4b. The relationship between heterogeneous networks and innovative opportunities is positively mediated by linear DM*.
*H4c. The relationship between heterogeneous networks and imitative opportunities is positively mediated by non-linear DM*.
*H4d. The relationship between heterogeneous networks and imitative opportunities is positively mediated by linear DM*.
*H4e. The relationship between homogeneous networks and innovative opportunities is positively mediated by non-linear DM*.
*H4f. The relationship between homogeneous networks and innovative opportunities is positively mediated by linear DM*.
*H4g. The relationship between homogeneous networks and imitative opportunities is positively mediated by non-linear DM*.
*H4h. The relationship between homogeneous networks and imitative opportunities is positively mediated by linear DM*.

**Figure 1:**
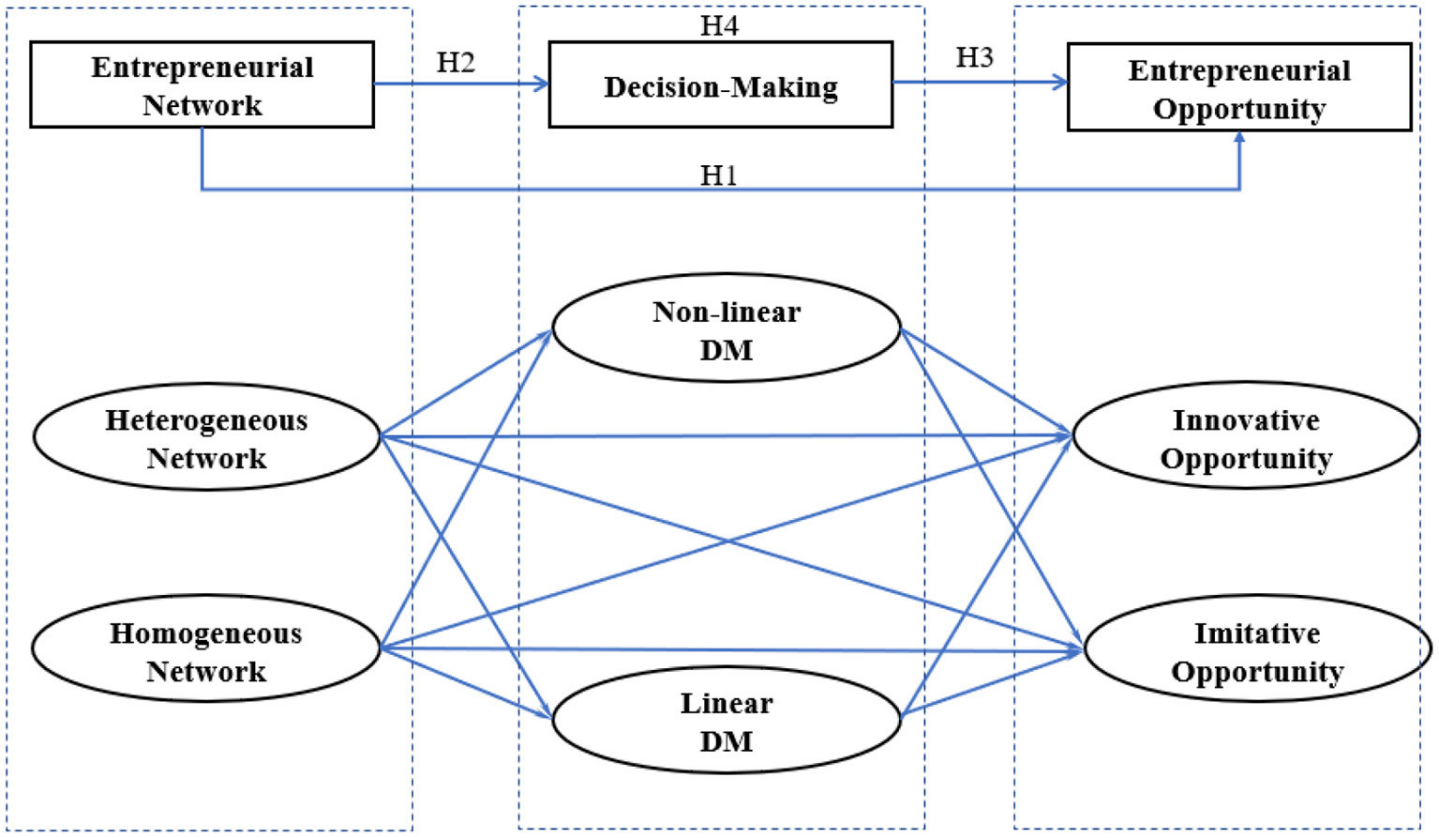
Conceptual framework.

Focusing on the mechanism of entrepreneurial opportunities, Figure 1 displays our mediation model that links entrepreneurial networks to entrepreneurial opportunities through decision-making.

## Methodology

### Sample and Data Description

This study used structured questionnaires to collect data from entrepreneurial firms that are located near the Yangtze River Delta region and have a high entrepreneurial activity index. The research objects are enterprise founders or co-founders of start-ups established within the last eight years. To ensure the questionnaire's reliability and validity, the survey content was revised based on previous studies. First, we selected 20 start-ups for semi-structured interviews and pre-tests and solicited opinions from five academic experts in the field of entrepreneurship to come up with the final questionnaire. The formal survey was carried out from December 2018 to February 2019, mainly through field distribution of paper questionnaires and peer-to-peer mobile-terminal-forwarding of the questionnaires online. The survey sample covered 26 urban areas in four provinces and cities in Zhejiang, Jiangsu, Shanghai, and Anhui. A total of 800 questionnaires were distributed, and 566 were collected. Questionnaires with responses indicating more than nine years of establishment and those with defective content were excluded. Finally, 512 valid questionnaires were obtained. The recovery and efficiency rates were 70.75% and 64.0%, respectively. Table 1 presents the descriptive statistics of the sample.

**Table 1:** The characteristics of the sample.

**Project**	**Category**	**Frequency**	**Percentage**	**Project**	**Category**	**Frequency**	**Percentage**
Gender	Male	405	79.1%	Industry category	Cars and parts	35	6.8%
	Female	107	20.9%		Communication electronics, computers, and internet	107	20.9%
Education	High school and below	97	18.9%		New materials, new energy, energy saving, and environmental protection	103	20.1%
	College	135	26.4%		Medical biological products	96	18.8%
	Bachelor	187	35.5%		Precision machinery	66	12.9%
	Master and Ph.D.	93	18.2%		Chemical, textile, and traditional manufacturing	67	13.1%
Company establishment period (year)	1–3	165	32.2%		Others	38	7.4%
	3–5	231	45.1%	Sales income (ten thousand yuan)	Below 100	30	5.9%
	5–8	116	22.7%		101–500	75	14.7%
Business size (number of people)	1–50	65	12.7%		501–1,000	122	23.8%
	51–100	111	21.7%		1,001–5,000	205	40.0%
	101–300	160	31.3%		More than 5,000	80	15.6%
	301–500	98	19.1%	Research area	Jiangsu	145	28.3%
	More than 500	78	15.2%		Zhejiang	188	36.7%
Number of startups	First venture	389	76.0%		Shanghai	77	15.1%
	Second & above	123	24.0%		Anhui	102	19.9%

### Common Method Bias

To minimize the influence of common method bias, time-lagged data collection was designed. The first round of surveys allowed respondents to answer the items related to the independent, mediator, and control variables. One week later, all the participants were asked to answer the items concerning the dependent variables. In addition, Harman's single-factor test was applied to detect the possibility of common method bias. The results showed that the extracted variables accounted for about 24.1% of the variance. Therefore, common method bias is not a serious concern for this study.

### Variables

Our study comprises three main variables and several control variables. Table 2 shows the three main variables in our model. These are classified into six sub-variables measured by five-point Likert scales.

**Table 2:** The reliability and validity of the measurement model.

**Variable**	**Dimension**	**Items**	**Factor loading**	**α**
Entrepreneurial network	Heterogeneous network	Maintain close relationships with different types of industries (main business) and actors with different nature	0.853	0.853
		Maintain close relationships with actors engaged in different research directions	0.745	
		Maintain close relationships with actors in different regions	0.823	
		Maintain close relationships with actors in different target markets	0.779	
		Maintain close relationships with actors of different cultures and ways of thinking	0.702	
	Homogeneous network	Close relationship with similar industry (main business) and actors with similar nature	0.832	0.876
		Keep a close relationship with actors engaged in similar research directions	0.712	
		Maintain close relationships with actors in similar areas	0.865	
		Maintain close relationships with actors in similar target markets	0.826	
		Keep a close relationship with actors who are close to one's culture and way of thinking	0.716	
Decision-making	Non-linear DM	When making career decisions, I mainly rely on my own feelings.	0.754	0.901
		Intuitive judgments are often correct when making large purchases or investment decisions.	0.788	
		When making major decisions, special attention is paid to the most direct physiological reactions such as tingling and chills in the bones.	0.677	
		The most important factor in changing your life (such as changing jobs, getting married, or a major relocation) is that it suits you.	0.879	
		When analysis and intuition are in conflict, give priority to intuition.	0.863	
	Linear DM	Mainly relying on logic when making career decisions	0.883	0.867
		Consider quantitative factors such as my age, budgetary needs, or future income when deciding to buy or invest	0.912	
		When making important decisions, I pay close attention to people that have sufficient professional knowledge to give me the same advice.	0.698	
		The most important factor in making a life-change decision is knowing that this change is based on objective, verifiable facts.	0.765	
		When analysis and intuition are in conflict, prioritize analytical reasoning.	0.814	
Entrepreneurial opportunity	Innovative opportunities	Products and services belong to a brand new market	0.855	0.788
		Provision of new products and services with new technologies (patents) and processes	0.802	
		Products and services require a high initial R&D investment	0.767	
		Products and services are less competitive in existing markets	0.675	
	Imitative opportunities	Products and services are minor improvements to existing technologies or processes	0.811	0.805
		Products and services are improvements in style, packaging, service, and so on.	0.752	
		Adjustments and improvements to existing marketing methods (such as prices, promotions, channels, etc.)	0.817	
		Products and services are more competitive in the existing market	0.677	

#### Dependent Variable: Entrepreneurial Opportunity

For an in-depth investigation of the nature of entrepreneurial opportunity in a dynamic environment, entrepreneurial opportunity is classified into two types: innovative opportunity (entrepreneurs develop new product/service/technology to create new markets) and imitative opportunity (entrepreneurs improve product/service/technology to adapt and extend the existing market). The question items are mainly based on previous literature (Holmén et al., 2007; Samuelsson and Davidsson, 2009).

#### Independent Variable: Entrepreneurial Network

In this study, entrepreneurial networks are divided into heterogeneous networks and homogeneous networks. The former is defined as the collaboration of different complementary firms, while the latter is a collaboration of similar firms. The question items were developed based on previous studies (Xin and Pearce, 1996; Möller and Halinen, 2000; Beckman and Haunschild, 2002; Wang et al., 2012).

#### Mediating Variable: Decision-Making

Decision-making is measured by non-linear DM (the use of internal feelings and intuition to process information) and linear DM (the preference for using external data and facts processed with rational/logical thinking). Following Vance et al. (2008) and Groves et al. (2011), five question items that measure non-linear and linear DM, respectively, were developed.

#### Control Variables

To control for the internal effect, four control variables (industry category dummy, entrepreneurial experience, firm size, and firm age) are included in the analysis, as they are likely to influence entrepreneurial opportunity.

## Analysis and Results

### Evaluation of the Research Model

The factor loadings and Cronbach's alpha of the items show that the scales have good reliability (see Table 2). The factor loading of all items is >0.6, and the Cronbach's alpha value of each variable is >0.7. Furthermore, the Kaiser-Meyer-Olkin (KMO) and Bartlett's test for sphericity are performed to check if the measured variables can be factorized efficiently. The results show that the KMO values of all variables are >0.75, while Bartlett's test of sphericity confirms the good quality of the items used for measuring the variables. Table 3 shows the means, standard deviations, and Pearson correlations of the key variables. The correlation coefficient of each variable is far <0.7, which supports further regression analysis to determine the causal relationship.

**Table 3:** Pearson's correlation matrix.

	**Mean**	**SD**	**1**	**2**	**3**	**4**	**5**	**6**	**7**	**8**	**9**	**10**
1. Entrepreneurial experience	1.36	0.53	1									
2. Industry category	3.03	0.63	−0.112	1								
3. Firm age	4.56	0.46	0.187	−0.302^*^	1							
4. Firm size	3.78	0.78	0.235^*^	−0.231	0.511^**^	1						
5. Heterogeneous network	3.83	0.58	0.462	0.293^**^	0.451	0.356^**^	1					
6. Homogeneous network	3.67	0.62	−0.230	−0.142	0.269^**^	0.220^**^	0.245^*^	1				
7. Non-linear DM	4.21	0.59	−0.324^**^	0.453	0.086	0.187	0.584^**^	0.342^**^	1			
8. Linear DM	4.06	0.66	0.163^**^	0.244	0.077^**^	0.324	0.230	0.287^**^	0.263^*^	1		
9. Innovative opportunity	3.29	0.77	0.422^**^	0.309^**^	0.178^*^	0.077	0.496^**^	0.362^**^	0.568^**^	0.526^**^	1	
10. Imitative opportunity	3.86	0.62	0.313^**^	0.183	0.201	0.181^**^	0.351^**^	0.473^**^	0.320^**^	0.653^**^	0.306^*^	1

* and ** *denote significance at the 5 and 1% levels, respectively*.

### Regression Analyses

We employ hierarchical regression analyses using SPSS 20.0 and use the criteria recommended by Hair et al. (2010). Table 4 shows the results of the hierarchical regression analyses conducted to estimate the effects of entrepreneurial network and decision-making on the entrepreneurial opportunity, as well as the impact of the entrepreneurial network on decision-making.

**Table 4:** The results of the regression analyses.

**Dependent variable**		**Innovative opportunity**		**Imitative opportunity**		**Non-linear DM**	**Linear DM**
		**Model 1**	**Model 2**	**Model 3**	**Model 4**	**Model 5**	**Model 6**
Control variables	Entrepreneurial experience	0.138	0.143^**^	0.157	0.088	0.168^**^	0.220
	Industry category	0.156^**^	0.094	0.116^**^	0.066^*^	0.083	0.101
	Firm age	−0.231	−0.412^*^	0.173	0.201^*^	−0.019	−0.326^**^
	Firm size	0.054^**^	0.134	0.251^**^	0.313	−0.056	−0.076
Independent variables	Heterogeneous network	0.613^**^		0.398		0.478^**^	0.411
	Homogeneous network	0.423		0.508^**^		0.375	0.605^**^
	Non-linear DM		0.566^**^		0.533		
	Linear DM		0.425		0.606^**^		
	R-squared	0.467	0.467	0.438	0.546	0.523	0.385
	F value	8.66	8.66	9.05	7.47	8.55	6.76
	Sig. (F)	0.003	0.003	0.000	0.000	0.000	0.000

* and ** *denote significance at the 5 and 1% levels, respectively*.

H1 predicts the positive influence of entrepreneurial networks on entrepreneurial opportunities. As Model 1 shows, the influence of heterogeneous networks on innovative opportunities is positive and significant (β = 0.613; *p* < 0.01); thus, H1a is supported. However, Model 3 indicates that H2b is not supported (β = 0.398; *p* > 0.05): there is no significant relationship between heterogeneous networks and imitative opportunities. H1c predicts a positive relationship between homogeneous networks and innovative opportunities. Similar to Model 1, the effect of homogeneous networks on innovative opportunities found to be positive, but it is insignificant (β = 0.423; *p* > 0.05). The result with respect to H4d indicates that homogeneous networks are positively linked to imitative opportunities (β = 0.508; *p* < 0.01); thus, H1d is supported. H1 is partially supported, specifically H1a and H1d. It can be seen that not all network types have a positive impact on entrepreneurial opportunities, and different networks have different effects on various ways of recognizing opportunities.

H2 predicts a positive relationship between entrepreneurial networks and decision-making. Model 5 indicates that heterogeneous networks have a positive effect on non-linear DM (β = 0.478; *p* < 0.01), thereby supporting H2a. However, Model 6 shows that the influence of heterogeneous networks on linear DM is positive but insignificant (β = 0.411; *p* > 0.05). The result for H2c indicates that the relationship between homogeneous networks and non-linear DM is positive but insignificant (β = 0.375; *p* > 0.05). Finally, H2d, which predicts that homogeneous networks are positively related to linear DM, is also supported (β = 0.605; *p* < 0.01). Accordingly, H2 is partially supported with the acceptance ofH2a and H2d. In short, the results show that interactions among entrepreneurship actors in networks that have different characteristics affect entrepreneurs' decision-making.

H3 is about the relationship between decision-making and entrepreneurial opportunities. Model 2 shows that non-linear DM has a positive effect on innovation opportunities (β = 0.566; *p* < 0.01), which supports H3a. Meanwhile, non-linear DM is found to have a positive but insignificant impact on innovation opportunities (β = 0.533; *p* > 0.05). By contrast, Model 4 indicates that the relationship between linear DM and imitative opportunities is positive but insignificant (β = 0.425; *p* > 0.05) and that the influence of linear DM on imitative opportunities is positive and significant (β = 0.606; *p* < 0.01). Therefore, H3 is partially supported by the acceptance of H3a and H3d. In short, the results show that the different decision-making styles of entrepreneurs have different effects on diverse types of entrepreneurial opportunities.

### Mediation Analyses

To investigate the mediating role of decision-making in the relationship between entrepreneurial networks and entrepreneurial opportunities, this study employs structural equation modeling supported by AMOS 20.0, following Hair et al. (2010) and Preacher and Hayes (2008). First, the fit of the research model is as follows: χ*2/df* = 1.762; *GFI* = 0.912; *CFI* = 0.930; *NFI* = 0.922; *RMR* = 0.023; and *RMSEA* = 0.047. Thus, it is proven that the model has a good fit and provides sufficient support for the results.

**Figure 2:**
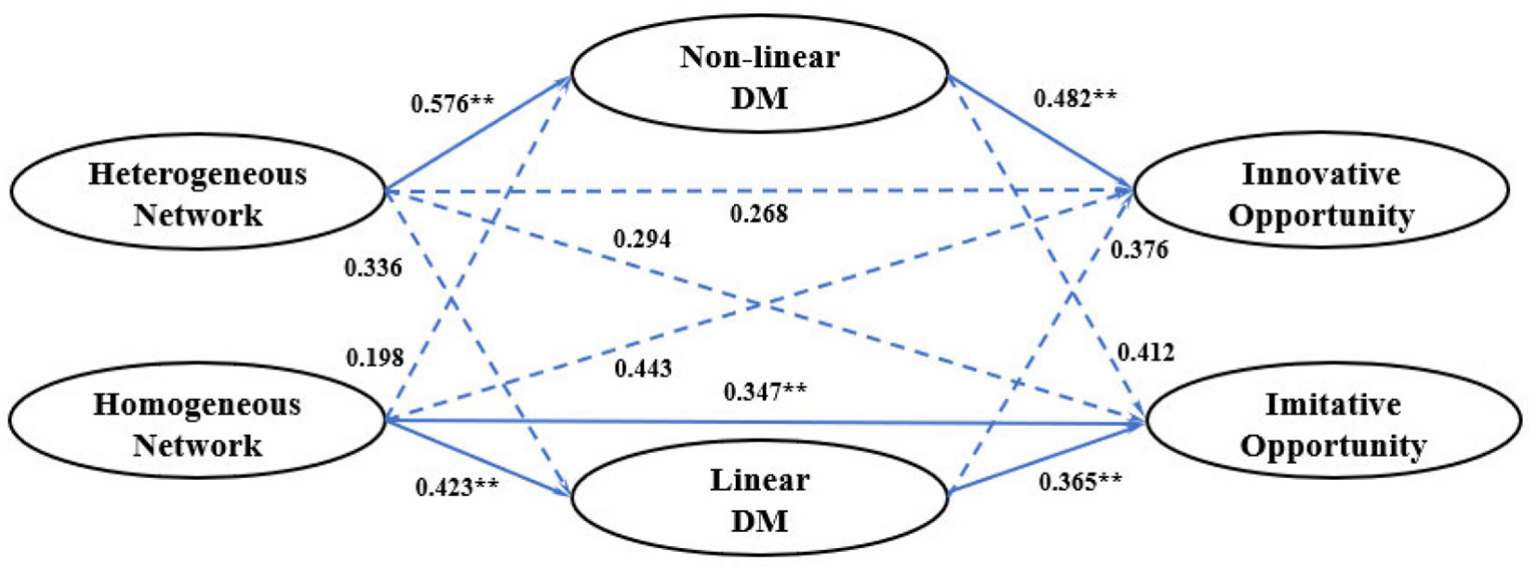
The results of the mediation analyses. (** denote significance at the 1% levels).

The results of the mediation analyses quantify H4 and show that the relationship between entrepreneurial networks and entrepreneurial opportunities is positively mediated by decision-making (see Figure 2). Moreover, the positive relationship between heterogeneous networks and innovative opportunities is insignificant after adding the mediator, non-linear DM. Furthermore, the indirect effect of heterogeneous networks on innovative opportunities via non-linear DM is positive and significant (β = 0.278; *p* < 0.01). Therefore, the results indicate that non-linear DM fully mediates the relationship between heterogeneous networks and innovative opportunities, which supports H4a. Moreover, the results show that linear DM plays a partial mediating role in the relationship between homogeneous networks and imitative opportunities. The indirect effect of homogeneous networks on imitative opportunities via linear DM is significantly positive (β = 0.154; *p* < 0.01). Hence, H4h is supported. Overall, the results indicate the mediating role of decision-making in the link between entrepreneurial networks and opportunities.

Finally, Table 5 presents a summary of the estimated results, showing that H1a, H1d, H2a, H2d, H3a, H4d, H4a, and H4h are supported while the other hypotheses are rejected.

**Table 5:** Summary of the estimated results.

**Path**	**Result**
H1a. Heterogeneous network → Innovative opportunity	Supported
H1b. Heterogeneous network → Imitative opportunity	Rejected
H1c. Homogeneous network → Innovative opportunity	Rejected
H1d. Homogeneous network → Imitative opportunity	Supported
H2a. Heterogeneous network → Non-linear DM	Supported
H2b. Heterogeneous network → Linear DM	Rejected
H2c. Homogeneous network → Non-linear DM	Rejected
H2d. Homogeneous network → Linear DM	Supported
H3a. Non-linear DM → Innovative opportunity	Supported
H3b. Non-linear DM → Imitative opportunity	Rejected
H3c. Linear DM → Innovative opportunity	Rejected
H3d. Linear DM → Imitative opportunity	Supported
H4a. Heterogeneous network → Non-linear DM → Innovative opportunity	Supported
H4b. Heterogeneous network → Linear DM → Innovative opportunity	Rejected
H4c. Heterogeneous network → Non-linear DM → Imitative opportunity	Rejected
H4d. Heterogeneous network → Linear DM → Imitative opportunity	Rejected
H4e. Homogeneous network → Non-linear DM → Innovative opportunity	Rejected
H4f. Homogeneous network → Linear DM → Innovative opportunity	Rejected
H4g. Homogeneous network → Non-linear DM → Imitative opportunity	Rejected
H4h. Homogeneous network → Linear DM → Imitative opportunity	Supported

## Discussion and Implications

Social media provides entrepreneurs with an opportunity to enlarge exposure to information within their business networks. Following previous studies on entrepreneurship, this study investigates the relationships among entrepreneurial networks, decision-making, and entrepreneurial opportunities in China and explores the mechanism of entrepreneurial opportunity formation in a dynamic environment, providing important insights for entrepreneurs.

### Major Findings

This study finds that heterogeneous networks have a positive effect on innovative opportunities, but homogeneous networks positively influence imitative opportunities. Heterogeneous networks provide numerous, non-redundant sources of information that help entrepreneurs acquire broad knowledge and different ideas and perspectives for improving decision-making. Heterogeneous networks offer an early frontier—where information may be conflicting and different interpretations are possible—which is advantageous in identifying and developing new ideas. Furthermore, heterogeneous actors introduce new knowledge and views to entrepreneurs, and they have multiple views on specific issues. This leads to discussions and exchanges of different views that eventually help in generating new ideas.

Homogeneous networks help entrepreneurs use the opportunities embedded in the business environment effectively. Entrepreneurs belonging to homogeneous networks are more likely to interact with other actors to share their experiences and their knowledge of the relevant market and service market methods, as well as provide suggestions to handle customer-related issues, and so on. This helps entrepreneurs design and adjust their marketing mix, understand and find opportunities, and make informed decisions about the associated risks. Imitative opportunities require entrepreneurs to search actively for asymmetric information that is embedded within homogeneous networks. In the pursuit of innovative opportunities, entrepreneurs can only partially benefit from the networks of actors that are similar to themselves. Since this process involves the creation of new knowledge, the prior knowledge of existing industries and markets is less helpful and may even be harmful to entrepreneurs that seek innovative opportunities (Campos et al., 2015). Our finding is consistent with Upson et al. (2017), who used data collected from women entrepreneurs in India. They suggested that entrepreneurs that operate in “discovery” contexts tend to participate in homogeneous networks, while entrepreneurs in “creation” contexts tend to participate in heterogeneous networks (Deng and Chen, 2021).

In addition, this study shows that heterogeneous networks positively affect non-linear DM, while homogeneous networks positively influence linear DM. The decision-making process for pursuing innovative opportunities is fraught with uncertainty, and heterogeneous networks help entrepreneurs develop flexible strategies for making changes when appropriate. In this sense, heterogeneous actors can help in the recruitment of human resources that have diverse knowledge bases to strengthen the opportunity-creation process. Finally, heterogeneous actors can measure what is deemed to be an “acceptable loss” based on different functions and perspectives—by judging the value of opportunities and by helping to evaluate the formation of opportunities. Furthermore, we find a positive relationship between homogeneous networks and linear DM. Linear thinking and linear DM are mostly influenced by the accumulation, inheritance, and spread of extant knowledge (Zhou and George, 2003). In other words, homogeneous networks help entrepreneurs focus on the current demand through the flexible use of professional knowledge, industrial experience, and market know-how. Overall, homogeneous networks contribute to entrepreneurs' linear DM.

The relationships between non-linear DM and innovative opportunities and between linear DM and imitative opportunities are found to be positive. Innovative opportunities require novel-thinking entrepreneurs that possess greater creativity and have access to rare new information. Non-linear thinking goes beyond existing knowledge and involves exploration of the unknown to obtain new ideas and viewpoints. Creativity in the non-linear thinking style and non-linear DM help in the creation of opportunities, as advocated by Schumpeter (Campos et al., 2015). However, linear DM is likely to lead to imitative opportunities, and we think that linear DM can help entrepreneurs correct market imperfections. Both non-linear DM and linear DM result from the entrepreneurs' cognitive processing of internal and external factors, but they show different characteristics and lead to inconsistent identification of entrepreneurial opportunities.

Non-linear DM fully mediates between heterogeneous networks and innovative opportunities, and linear DM partially mediates between homogeneous networks and imitative opportunities. Our findings provide a solution to the inherent mechanism of entrepreneurial networks and entrepreneurial opportunity identification. If entrepreneurs want to identify entrepreneurial opportunities quickly and accurately, they must start by building entrepreneurial networks and becoming familiarized with the business environment. The use of information and resources within homogeneous networks can help entrepreneurs with linear DM to identify and utilize imitative opportunities in the market. Meanwhile, heterogeneous networks are not highly correlated with knowledge and thus, are not helpful in identifying innovative opportunities, unless the actors exert non-linear DM to deconstruct, connect, and reconstruct the acquired information and knowledge. Therefore, non-linear DM serves as a “central processing unit” in the process of innovative opportunity recognition.

### Theoretical Contributions

This study makes several theoretical contributions. It goes beyond investigating the impact of endogenous and exogenous perspectives on entrepreneurial opportunity identification—by determining the relationship between networks and opportunities within entrepreneurial firms. This study also proposes a new entrepreneurial opportunity identification model based on organic integration of both external (entrepreneurial networks) and internal (decision-making) factors by developing a mediation model among entrepreneurial networks, decision-making, and opportunities. Overall, we provide an integrated framework that examines the role of decision-making in the relationship between entrepreneurial networks and opportunities.

In addition, our findings contribute to literature on entrepreneurial opportunities by determining the impact of different networks on diverse opportunities. Not all networks are effective under different contexts, and only those that match the entrepreneurs' personal characteristics, abilities, and goals are valuable (Shu et al., 2018). This study explores how entrepreneurs develop opportunities using social resources and thereby provides a new perspective for understanding what is considered a “high-quality network” in the context of social media. Being of “high quality” means that a network is capable of presenting opportunities that entrepreneurs seek, and this matching relationship is a kind of resource that cannot be imitated. Entrepreneurship constitutes the backbone of business network formation and development, which relates to the quantity and quality of the actors: as a relevant business progresses and constantly changes, the range of the network gradually changes from small to large, and the boundary also transforms from undefined to clear and relatively fixed. Therefore, networks are constantly and dynamically optimizing, and the formation and development of entrepreneurial networks are the result of the interactions among different actors. This paper extends prior perspectives on entrepreneurial opportunity by discussing the use of heterogeneous and homogeneous networks, thereby providing new insights and trends for the study of entrepreneurial networks.

This study contributes to understanding of the formation mechanisms of opportunity as defined by both Schumpeter and Kirzner. Importantly, this study provides empirical evidence of the mediating role of different decision-making styles in the relationship between entrepreneurial networks and opportunities. This study extends the findings of Maine (2015), who identified that various decision-making styles may result in opportunity creation and recognition. Specifically, we find that non-linear DM is positively related to innovative opportunities, and linear DM is positively related to imitative opportunities. Furthermore, the contribution of entrepreneurial networks to opportunity identification depends on the degree of matching among the entrepreneurial networks, decision-making, and opportunities. Specifically, the formation of innovative opportunities depends on the link between heterogeneous networks and linear DM. Meanwhile, imitative opportunities require the interface between homogeneous networks and linear DM. Scholars such as de Jong and Marsili (2015) have stated that “opportunities are highly heterogeneous,” and our study supports this position, as it has demonstrated that homogeneous networks and linear DM can also lead to the identification of entrepreneurial opportunities.

### Practical Implications

Our study has several implications for both entrepreneurs and policymakers in emerging economies. First, for entrepreneurs in a dynamic environment, matching the network, decision-making style, and opportunity is very important. Entrepreneurs need to understand the connotation and category of entrepreneurial opportunities and choose suitable entrepreneurial goals according to their own conditions and the development level of the target region. To achieve their goals, entrepreneurs should actively select the appropriate partners and thereby build high-quality networks. This study demonstrates the impact of different decision-making styles in the process of entrepreneurial opportunity formation. Entrepreneurs should constantly hone their mode of thinking; enhance their restructuring ability; and acquire information, knowledge, and business networks that match the entrepreneurial opportunities they are seeking, and thereby transform networks into opportunities and improve the growth of start-ups.

For policymakers, this study provides valuable insights about supporting innovative and imitative entrepreneurship. On the one hand, in consideration of the types of entrepreneurial opportunities, entrepreneurial motives, and needs of entrepreneurs, local governments should put forward targeted entrepreneurial policies for different categories of entrepreneurship. For ordinary entrepreneurs and regions with relatively backward economic development, imitation opportunities can be found in the consumers' demands. This represents a low-risk, flexible, and relatively easy way to realize entrepreneurial goals that lead to relatively stable entrepreneurial income. The government also needs to reformulate the relevant policies according to specific circumstances to guide entrepreneurs in their pursuit of imitative and innovative opportunities—by, for example, establishing a multi-level innovative venture capital market system, expanding financing channels, building risk diversification mechanisms, improving the talent market, and further introducing an incentive system for transforming scientific and technological achievements to stimulate innovative entrepreneurship (de Jong and Marsili, 2015). Thereafter, the government should give full play to the demonstration effect of economically developed regions. Finally, the government also needs to improve the service system of innovative entrepreneurship. Governmental agencies need to understand the actual needs of entrepreneurs and come up with an innovative service model to provide entrepreneurial information.

### Limitations and Directions for Future Research

Although this study provides important insights into entrepreneurship, this work is not free of limitations. The first limitation concerns the study's cross-sectional design, whereby survey data were collected from Chinese entrepreneurial firms. Panel data is thus required to determine the relationships among entrepreneurial networks, decision-making, and opportunities. Second, the study is limited to the investigation of the influence of both networks and decision-making on entrepreneurial opportunities and fails to consider the interaction effect of the two independent variables. Third, the study investigates Chinese entrepreneurs' behaviors, and the findings may differ from those in other emerging countries. Finally, this study is exploratory research and future studies need to test these results through a multi-method investigation.

## Data Availability Statement

The raw data supporting the conclusions of this article will be made available by the authors, without undue reservation.

## Ethics Statement

The studies involving human participants were reviewed and approved by Gachon University Ethics Committee. The patients/participants provided their written informed consent to participate in this study. Written informed consent was obtained from the individual(s) for the publication of any potentially identifiable images or data included in this article.

## Author Contributions

All authors listed have made a substantial, direct and intellectual contribution to the work, and approved it for publication.

## Conflict of Interest

The authors declare that the research was conducted in the absence of any commercial or financial relationships that could be construed as a potential conflict of interest.

## References

[B1] Adner R. Kapoor R. (2010). Value creation in innovation ecosystems: how the structure of technological interdependence affects firm performance in new technology generations. Strategic Manage. J. 31, 306–333. 10.1002/smj.821

[B2] Aldrich H. E. Cliff J. E. (2003). The pervasive effects of family on entrepreneurship: toward a family embeddedness perspective. J. Bus. Venturing 18, 573–596. 10.1016/S0883-9026(03)00011-9

[B3] Ardichvili A. Cardozo R. Ray S. (2003). A theory of entrepreneurial opportunity identification and development. J. Bus. Venturing 18, 105–123. 10.1016/S0883-9026(01)00068-4

[B4] Beckman C. M. Haunschild P. R. (2002). Network learning: the effects of partners' heterogeneity of experience on corporate acquisitions. Admin. Sci. Quart. 47, 92–124. 10.2307/3094892

[B5] Campos H. M. Parellada F. S. Quintero M. R. Valenzuela F. A. A. (2015). Creative thinking style and the discovery of entrepreneurial opportunities in startups. Revista de Negócios 20, 3–12. 10.7867/1980-4431.2015v20n1p3-12

[B6] Cantù C. (2018). Discovering the collective entrepreneurial opportunities through spatial relationships. IMP J. 12, 276–295. 10.1108/IMP-05-2017-0033

[B7] Cardon M. S. Post C. Forster W. R. (2017). Team entrepreneurial passion: its emergence and influence in new venture teams. Acad. Manage. Rev. 42, 283–305. 10.5465/amr.2014.0356

[B8] Chen M. (2019). The impact of expatriates' cross-cultural adjustment on work stress and job involvement in the high-tech industry. Front. Psychol. 10:2228. 10.3389/fpsyg.2019.02228 31649581 PMC6794360

[B9] Dane E. Pratt M. G. (2007). Exploring intuition and its role in managerial decision making. Acad. Manage. Rev. 32, 33–54. 10.5465/amr.2007.23463682

[B10] Davidsson P. (2015). Entrepreneurial opportunities and the entrepreneurship nexus: a re-conceptualization. J. Bus. Venturing 30, 674–695. 10.1016/j.jbusvent.2015.01.002

[B11] Davidsson P. Honig B. (2003). The role of social and human capital among nascent entrepreneurs. J. Bus. Venturing 18, 301–331. 10.1016/S0883-9026(02)00097-6

[B12] de Jong J. P. J. Marsili O. (2015). The distribution of Schumpeterian and Kirznerian opportunities. Small Bus. Econ. 41, 19–35. 10.1007/s11187-014-9585-1

[B13] Deng X. Guo X. Wu Y. J. Chen M. (2021). Perceived environmental dynamism promotes entrepreneurial team member's innovation: explanations based on the uncertainty reduction theory. Int. J. Environ. Res. Public Health 18:2033. 10.3390/ijerph18042033 33669732 PMC7921965

[B14] Di Muro P. Turner J. R. (2018). Entrepreneurial opportunity pursuit through business model transformation: a project perspective. Int. J. Proj. Manage. 36, 968–979. 10.1016/j.ijproman.2018.07.001

[B15] Drummond C. McGrath H. O'Toole T. (2018). The impact of social media on resource mobilisation in entrepreneurial firms. Ind. Marketing Manage. 70, 68–89. 10.1016/j.indmarman.2017.05.009

[B16] Feng B. Chen M. (2020). The impact of entrepreneurial passion on psychology and behavior of entrepreneurs. Front. Psychol. 11:1733. 10.3389/fpsyg.2020.01733 32793066 PMC7385187

[B17] Foss N. J. Lyngsie J. Zahra S. A. (2013). The role of external knowledge sources and organizational design in the process of opportunity exploitation. Strategic Manage. J. 34, 1453–1471. 10.1002/smj.2135

[B18] Groves K. Vance C. Choi D. (2011). Examining entrepreneurial cognition: an occupational analysis of balanced linear and nonlinear thinking and entrepreneurship success. J. Small Bus. Manage. 49, 438–466. 10.1111/j.1540-627X.2011.00329.x

[B19] Groves K. Vance C. Paik Y. (2008). Linking linear/nonlinear thinking style balance and managerial ethical decision-making. J. Bus. Ethics 80, 305–325. 10.1007/s10551-007-9422-4

[B20] Hair J. F. Black W. C. Babin B. J. Anderson R. E. (2010). Multivariate Data Analysis: Global Edition. Upper Saddle River, NJ: Pearson Higher Education.

[B21] Hoang H. Antoncic B. (2003). Network-based research in entrepreneurship: a critical review. J. Bus. Venturing 18, 165–187. 10.1016/S0883-9026(02)00081-2

[B22] Holmén M. Magnusson M. McKelvey M. (2007). What are innovative opportunities? Ind. Innov. 14, 27–45. 10.1080/13662710601130830

[B23] Jansen R. J. Curşeu P. L. Vermeulen P. A. Geurts J. L. Gibcus P. (2013). Information processing and strategic decision-making in small and medium-sized enterprises: the role of human and social capital in attaining decision effectiveness. Int. Small Bus. J. 31, 192–216. 10.1177/0266242611406762

[B24] Kahneman D. (2003). A perspective on judgment and choice: mapping bounded rationality. Am. Psychol. 58:697. 10.1037/0003-066X.58.9.697 14584987

[B25] Kamm J. B. Nurick A. J. (1993). The stages of team venture formation: a decision-making model. Entrep. Theory Pract. 31, 17–27. 10.1177/104225879301700202

[B26] Kirzner I. M. (1973). Entrepreneurship and economic development. New York, NY.

[B27] Kirzner I. M. (1997). Entrepreneurial discovery and the competitive market process: an austrian approach. J. Econ. Lit. 35, 60–85.

[B28] Maine E. Pek-Hooi S. Santos N. D. (2015). The role of entrepreneurial decision-making in opportunity creation and recognition. Technovation 39, 53–72. 10.1016/j.technovation.2014.02.007

[B29] McMullen J. S. Dimov D. (2013). Time and the entrepreneurial journey: the problems and promise of studying entrepreneurship as a process. J. Manage. Stud. 50, 1481–1512. 10.1111/joms.12049

[B30] McMullen J. S. Shepherd D. A. (2006). Entrepreneurial action and the role of uncertainty in the theory of the entrepreneur. Acad. Manage. Rev. 31, 132–152. 10.5465/amr.2006.19379628

[B31] Möller K. Halinen A. (2000). Relationship marketing theory: its roots and direction. J. Market Manage. 16, 29–54. 10.1362/026725700785100460

[B32] Nieto M. J. Santamaría L. (2007). The importance of diverse collaborative networks for the novelty of product innovation. Technovation 27, 367–377. 10.1016/j.technovation.2006.10.001

[B33] Ozgen E. Baron R. A. (2007). Social sources of information in opportunity recognition: effects of mentors, industry networks, and professional forums. J. Bus. Venturing 22, 174–192. 10.1016/j.jbusvent.2005.12.001

[B34] Petuskiene E. Glinskiene R. (2011). Entrepreneurship as the basic element for the successful employment of benchmarking and business innovations. Inz Ekon. 22, 69–77. 10.5755/j01.ee.22.1.220

[B35] Preacher K. J. Hayes A. F. (2008). Asymptotic and resampling strategies for assessing and comparing indirect effects in multiple mediator models. Behav. Res. Methods 40, 879–891. 10.3758/BRM.40.3.879 18697684

[B36] Rosenbusch N. Brinckmann J. Bausch A. (2011). Is innovation always beneficial? a meta-analysis of the relationship between innovation and performance in SMEs. J. Bus. Venturing 26, 441–457. 10.1016/j.jbusvent.2009.12.002

[B37] Ruef M. Aldrich H. E. Carter N. M. (2003). The structure of founding teams: homophily, strong ties, and isolation among US entrepreneurs. Am. Sociol. Rev. 68, 195–222. 10.2307/1519766

[B38] Samuelsson M. Davidsson P. (2009). Does venture opportunity variation matter? investigating systematic process differences between innovative and imitative new ventures. Small. Bus. Econ. 33, 229–255. 10.1007/s11187-007-9093-7

[B39] Schumpeter J. A. (1934). Change and the Entrepreneur. Essays of JA Schumpeter.

[B40] Shane S. Nicolaou N. (2015). Creative personality, opportunity recognition and the tendency to start businesses: a study of their genetic predispositions. J. Bus. Venturing 30, 407–419. 10.1016/j.jbusvent.2014.04.001

[B41] Shane S. Venkataraman S. (2000). The promise of entrepreneurship as a field of research. Acad. Manage. Rev. 25, 217–226. 10.5465/amr.2000.2791611

[B42] Shane S. A. (2003). A General Theory of Entrepreneurship: The Individual-Opportunity Nexus. Cheltenham: Edward Elgar Publishing.

[B43] Shu R. Ren S. Zheng Y. (2018). Building networks into discovery: the link between entrepreneur network capability and entrepreneurial opportunity discovery. J. Bus. Res. 85, 197–208. 10.1016/j.jbusres.2017.12.048

[B44] Suddaby R. Bruton G. D. Si S. X. (2015). Entrepreneurship through a qualitative lens: insights on the construction and/or discovery of entrepreneurial opportunity. J. Bus. Venturing 30, 1–10. 10.1016/j.jbusvent.2014.09.003

[B45] Tamasy C. (2006). Determinants of regional entrepreneurship dynamics in contemporary Germany: a conceptual and empirical analysis. Reg. Stud. 40, 365–384. 10.1080/00343400600612137

[B46] Upson J. W. Damaraju N. L. Anderson J. R. Barney J. B. (2017). Strategic networks of discovery and creation entrepreneurs. Eur. Manag. J. 35, 198–210. 10.1016/j.emj.2017.01.001

[B47] Vance C. Zell D. Groves K. (2008). Considering individual linear/non-linear thinking style and innovative corporate culture. Int. J. Organ. Anal. 16, 232–248. 10.1108/19348830810944684

[B48] Vance C. M. Groves K. S. Paik Y. Kindler H. (2007). Understanding and measuring linear–non-linear thinking style for enhanced management education and professional practice. Acad. Manag. Learn. Edu. 6, 167–185. 10.5465/amle.2007.25223457

[B49] Wang K. Y. Wang Y. Huang K.-P. Deng J. (2012). Heterogeneous networks and resource acquisition of SMEs in emerging economies. Qual. Quant. 46, 1643–1657. 10.1007/s11135-011-9472-7

[B50] Xin K. K. Pearce J. L. (1996). Guanxi: connections as substitutes for formal institutional support. Acad. Manage. J. 39, 1641–1658. 10.2307/257072

[B51] Zhang Y. Wang Y. Wenhong Z. Liuying F. (2010). The impact of entrepreneurial thinking system on risk-taking propensity and entrepreneurial behavior. J. Chin. Entrepreneurship 2, 165–174. 10.1108/17561391011051144

[B52] Zhou J. George J. M. (2003). Awakening employee creativity: the role of leader emotional intelligence. Leadership Quart. 14, 545–568. 10.1016/S1048-9843(03)00051-1

